# Atrial fibrillation is not an independent determinant of 28-day mortality among critically III sepsis patients

**DOI:** 10.1186/s12871-023-02281-z

**Published:** 2023-10-06

**Authors:** Weiping Wang, Yujiang Dong, Qian Zhang, Hongmei Gao

**Affiliations:** 1 Department of Cardiology, Sunshine Union Hospital, Weifang, 261072 Shandong China; 2 Department of Cardiology, The Second Affiliated Hospital of Shandong University of Traditional Chinese Medicine, Jinan, 250001 Shandong China; 3 Shandong University of Traditional Chinese Medicine, Jinan, 250014 Shandong China

**Keywords:** Atrial fibrillation, Sepsis, Propensity score matching, Medical Information Mart for Intensive Care IV database

## Abstract

This study was conducted to investigate the relationship between atrial fibrillation and the clinical prognosis of patients with sepsis in intensive care unit. A total of 21,538 sepsis patients were enrolled in the study based on the Medical Information Mart for Intensive Care IV database, of whom 6,759 had AF. Propensity score matching was used to compare the clinical characteristics and outcomes of patients with and without AF. Besides, the inverse probability of treatment weighting, univariate and multivariate Cox regression analyzes were performed. Of the 21,538 patients, 31.4% had AF. The prevalence of AF increased in a step-by-step manner with growing age. Patients with AF were older than those without AF. After PSM, 11,180 patients remained, comprising 5,790 matched pairs in both groups. In IPTW, AF was not associated with 28-day mortality [hazard ratio (HR), 1.07; 95% confidence interval (CI), 0.99–1.15]. In Kaplan-Meier analysis, it was not observed difference of 28-day mortality between patients with and without AF. AF could be associated with increased ICU LOS, hospital LOS and need for mechanical ventilation; however, it does not remain an independent short-term predictor of 28-day mortality among patients with sepsis after PSM with IPTW and multivariate analysis.

**Supplementary Information:**

The online version contains supplementary material available at 10.1186/s12871-023-02281-z.

## Introduction

Sepsis has become one of the main causes of morbidity and mortality in intensive care units (ICUs). It has been reported that sepsis affects over 1 million hospitalized patients each year in the United States [1]. Sepsis accounts for approximately 30 to 50% of hospital deaths [2, 3], and the mortality rate of sepsis is significantly higher than that for other common acute diseases [4, 5]. AF is not only the most common and clinically relevant supraventricular arrhythmia encountered by physicians in daily clinical life [6] but also the most common persistent arrhythmia in critically ill patients [7–9]. AF complicates the course of disease and increases mortality in critically ill patients [9, 10]. AF is closely related to sepsis and is the most common arrhythmia in critically ill patients with sepsis [11]. Sepsis often induces new-onset AF. It has been reported that hospitalized patients with sepsis have a 6-fold higher risk of new-onset AF than hospitalized patients without sepsis [12]. The occurrence of new-onset AF during sepsis is associated with high morbidity and mortality [13–20]. The mechanisms of new-onset AF during sepsis are unclear and may involve the systemic release of proinflammatory cytokines, high levels of circulating stress hormones, autonomic dysfunction, intravascular volume shifts and cardiovascular compromise [13, 21, 22]. Although most studies showed that the development of AF was associated with increased mortality in patients with sepsis, these studies primarily included patients with new-onset AF who were not critically ill and usually had an imbalance in the baseline characteristics between the AF group and the group without AF. In addition, some studies reported that AF could not be considered a factor affecting increased mortality in patients with sepsis [23, 24].

This discrepancy might be because of differences in settings or study designs. A more reliable result that AF is an independent risk factor for mortality can be obtained using propensity score matching [25]. Hence, in the present retrospective study, PSM using detailed clinical data obtained from the Multiparameter Intelligent Monitoring in Intensive Care (MIMIC-IV) database was performed to investigate the relationship between AF and the characteristics of patients with sepsis admitted to the ICU and patient outcomes, including 28-day mortality, ICU length of stay (LOS), hospital LOS, and the need for mechanical ventilation.

## Materials and methods

### Source of data

This was a longitudinal, single-centre, retrospective cohort study based on the MIMIC-IV database (version 1.0), which is a large, comprehensive and openly accessible critical care database that comprises data from 53,150 critically ill patients who visited the Beth Israel Deaconess Medical Center in the United States from 2008 to 2019 [26, 27]. The MIMIC-IV database was established by the Massachusetts Institute of Technology and Beth Israel Deaconess Medical Center, and the ethical approval statement and informed consent were not required for this study because all patient data in the database were anonymized. After successfully completing the Collaborative Institutional Training Initiative examination (Certification Number 46,543,547 for author Weiping Wang), we obtained approval to access the MIMIC-IV database for data extraction. This cohort study was conducted according to the Declaration of Helsinki (as revised in 2013) [28].

### Inclusion and exclusion criteria

**Fig. 1:**
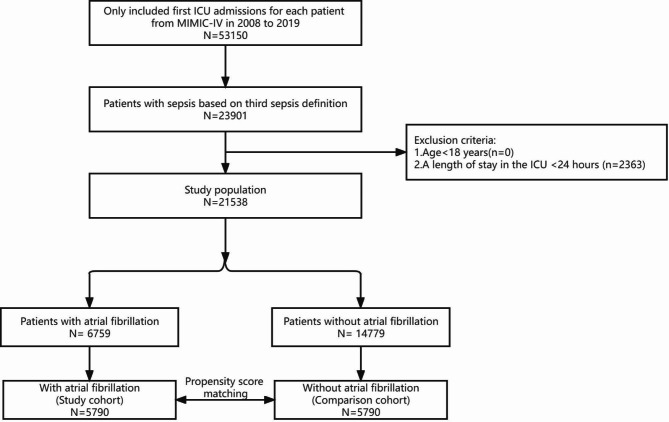
Flow diagram of the study. ICU: Intensive Care Unit

In this study, patients in the MIMIC-IV database who were diagnosed with sepsis at their first ICU admission during the period 2008–2019 were included. The diagnosis of sepsis was made in accordance with the Sepsis-3 guidelines [29]; sepsis was diagnosed in patients with at least one suspected site of infection and an acute increase in the Sequential Organ Failure Assessment (SOFA) score (scores ≥ 2). The diagnosis of AF was made using ICD diagnosis codes (ICD-9 code for AF = 42,731 and ICD-10 codes for AF = I48, I480, I481, I4811, I480, I4819, I482, I4820, I4821, I489, and I4891). The exclusion criteria were as follows: (1) age < 18 years and (2) an ICU length of stay < 24 h. A flow diagram of patient inclusion and exclusion is shown in Fig. 1.

### Data extraction

We used Structured Query Language with PostgreSQL 14.0 for data extraction. Data on the following were extracted: demographic information (e.g., age, sex and race), vital signs (e.g., mean arterial pressure [MAP], respiratory rate, heart rate and body temperature within 24 h of ICU admission), comorbidities (e.g., myocardial infarct, congestive heart failure, hypertension, hyperlipidaemia, peripheral vascular disease, cerebrovascular disease, dementia, chronic pulmonary disease, rheumatic disease, renal disease, peptic ulcer disease, mild liver disease, severe liver disease, diabetes and malignant cancer), laboratory parameters (e.g., haemoglobin level; leukocyte count [WBCs]; platelet count; the levels of blood urea nitrogen, blood creatinine, glucose, potassium, and sodium; pH; partial pressure of oxygen [pO_2_]; partial pressure of carbon dioxide [pCO_2_]; and partial thromboplastin time [ptt]) and scores (e.g., the SOFA score and Acute Physiology Score III [APS III]). There were 18 categories of medical conditions in the Charlson Comorbidity Index (CCI) identified in the patients’ medical records (Supplementary Table 1). If there were multiple results for the above mentioned indicators within 24 h, the worst value was recorded and used for the analysis.

### Management of missing data

In the present study, missing data were processed according to the missing percentage. Bilirubin, lactate and albumin data had more than 20% missing values and were excluded from the study. Correspondingly, multiple imputation was used to impute variables with less than 20% missing values [30, 31]. Details of missing data are shown in Supplementary Table 2.

### Outcomes

The main outcome was mortality at 28 days after the date of ICU admission. The secondary outcomes include the time of mechanical ventilation after ICU admission, ICU length of stay (LOS), and hospital LOS.

### Statistical analysis

Descriptive analysis was applied to all participants’ data. Data are presented as the total number and proportions (%) for categorical variables and as the mean ± standard deviation (normal distributions) or interquartile range (IQR) and median (skewed distributions) for continuous variables. For baseline characteristics analysis, significant differences between variables were tested with the chi-square test or Fisher’s exact test for categorical variables, with the t test or one-way ANOVA for normally distributed continuous variables, and with the Kruskal‒Wallis test for continuous variables with an asymmetric distribution.

Propensity score matching (PSM) was used to minimize the imbalance in baseline characteristics between the AF group and the group without AF. PSM was performed using a 1:1 nearest neighbour matching algorithm with a calliper width of 0.2 [32]. The following preoperative variables were used to generate the propensity score: age, sex, race, MAP, respiratory rate, heart rate, body temperature, MI, CHF, hypertension, hyperlipidaemia, PVD, cerebrovascular disease, dementia, chronic pulmonary disease, rheumatic disease, renal disease, peptic ulcer disease, mild liver disease, severe liver disease, diabetes, malignant cancer, haemoglobin, WBC count, platelet count, urea nitrogen level, creatinine level, glucose level, potassium level, sodium level, pH, pO_2_, pCO_2_, ptt, SOFA score, APS III and CCI score. The degree of PSM was examined by the standardized mean difference (SMD), and a value of less than 0.1 was considered acceptable. The matched data were used to compare both major and secondary outcomes. Sensitivity analysis was performed by removing patients with new-onset AF (Supplementary Table 3).

Using the estimated propensity scores as weights, an inverse probability of treatment weighting (IPTW) model was applied to generate a weighted cohort [33]. Then, the propensity score was adjusted by univariable Cox and multivariate proportional hazards regression. We also applied a univariable Cox proportional hazards regression model with the robust variance estimator to calculate the hazard ratio (HR) for mortality and performed Kaplan‒Meier and log-rank analyses to determine 28-day survival curves. All analyses were carried out with the statistical software packages SPSS 25 and Free Statistics software version 1.4. P < 0.05 was considered statistically significant.

## Results

### Patient characteristics

**Fig. 2:**
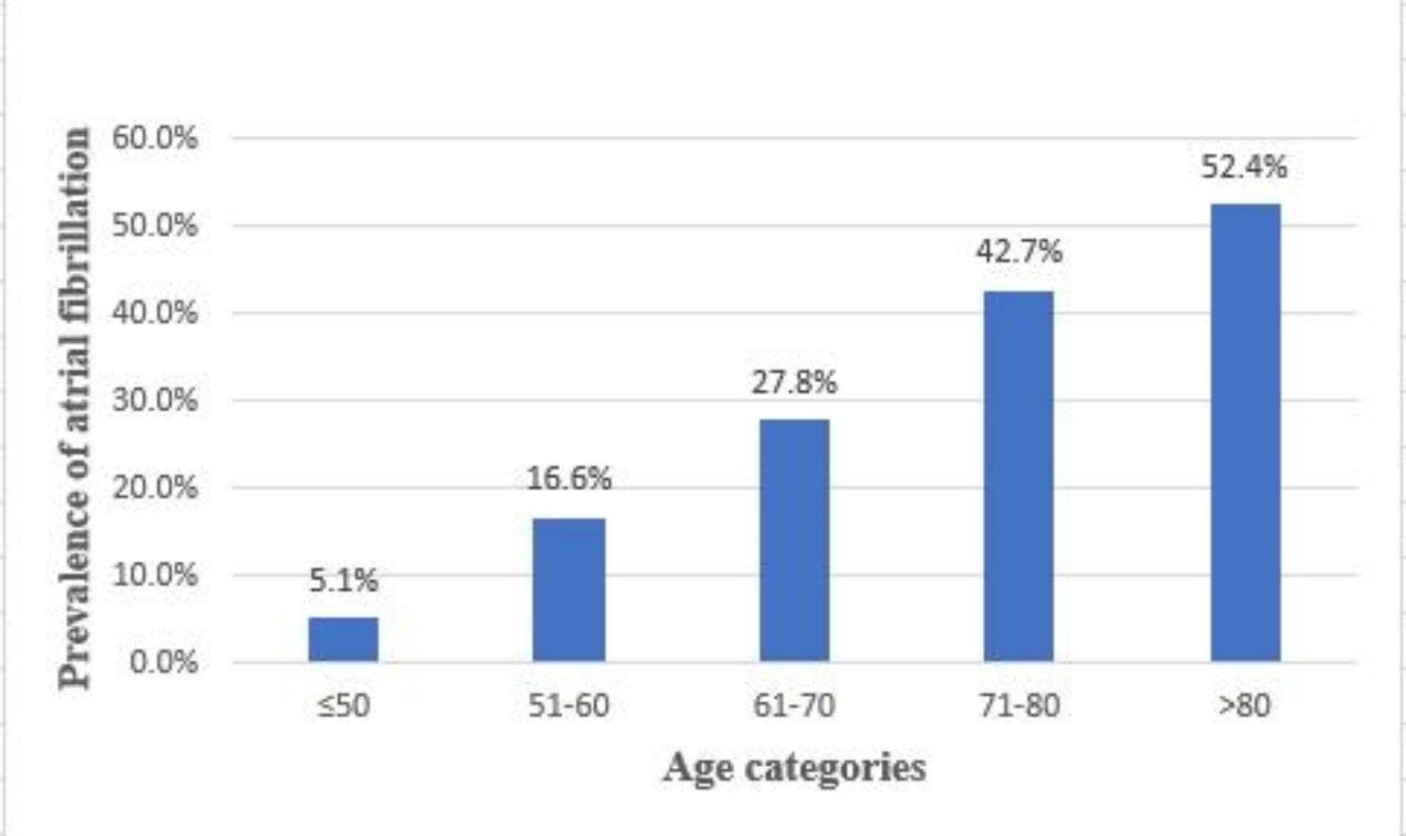
Prevalence of atrial fibrillation

A total of 21,538 sepsis patients were ultimately enrolled in the cohort study, including 6,759 patients with AF and 14,779 patients without AF (Fig. 1). There were 78 new-onset AF patients without a history of AF (0.53%, 78/14, 857). The baseline characteristics of the patients are presented in Table 1. Before PSM, there were significant differences in the covariates in Table 1, in addition to the respiratory rate and peptic ulcer disease, between the two groups. The patients in the AF group were more likely to be older than those in the non-AF group (76.1 vs. 63.6; p < 0.001). The prevalence of AF increased in a step-by-step manner with increasing age, from 5.1% in those younger than 50 years to 52.4% in those older than 80 years (Fig. 2). The AF group had a higher CCI score (6.0 vs. 5.0; p < 0.001), a higher APS III (52.0 vs. 49.0; p < 0.001) and had more comorbidities, including myocardial infarction (22.8% vs. 14.7%; p < 0.001), congestive heart failure (46.8% vs. 20.2%; p < 0.001), hypertension (45.2% vs. 42.1%; p < 0.001), hyperlipidaemia (45.0% vs. 31.9%; p < 0.001), peripheral vascular disease (16.5% vs. 10.0%; p < 0.001) cerebrovascular disease (16.2% vs. 13.8%; p < 0.001), dementia (5.3% vs. 3.9%; p < 0.001), chronic pulmonary disease (30.5% vs. 23.6%; p < 0.001), rheumatic disease (4.0% vs. 3.4%;p < 0.001), renal disease (28.7% vs. 17.4%; p < 0.001) and diabetes (32.6% vs. 28.7%; p < 0.001) (Table 1).

**Table 1:** Baseline characteristics before and after propensity score matching

Characteristics	Before matching				After matching			
	Non-AF group (n = 14,779)	AF group (n = 6759)	SMD	P value	Non-AF group (n = 5790)	AF group (n = 5790)	SMD	p value
Female, n (%)	6286 (42.5)	2733 (40.4)	< 0.1	0.004	2356 (40.7)	2378 (41.1)	< 0.1	0.691
Age (years)	63.6(52.5, 74.6)	76.1(67.4, 83.7)	0.864	< 0.001	74.5 (65.5, 82.8)	74.2 (66.0, 82.0)	< 0.1	0.149
Ethnicity, n (%)			0.209	< 0.001			< 0.1	0.869
White	9481 (64.2)	4924 (72.9)			4165 (71.9)	4158 (71.8)		
Black	1351 (9.1)	343 (5.1)			332 (5.7)	331 (5.7)		
Asian	445 (3)	164 (2.4)			134 (2.3)	148 (2.6)		
Other	3502 (23.7)	1328 (19.6)			1159 (20)	1153 (19.9)		
Heart rate (bpm)	85.7(75.8, 97.5)	84.2(75.1, 96.4)	< 0.1	< 0.001	83.9(75.1, 95.1)	83.9(74.8, 95.6)	< 0.1	0.836
MAP (mmHg)	76.1(70.4, 83.2)	74.0(68.9, 80.1)	0.238	< 0.001	74.4(69.0, 80.7)	74.3(69.1, 80.4)	< 0.1	0.945
Respiratory rate (bpm)	18.9(16.7, 22.0)	19.1(16.8, 21.9)	< 0.1	0.084	18.9(16.8, 21.9)	19.0(16.8, 21.9)	< 0.1	0.674
Temperature (°C)	37.4(37.0, 38.0)	37.2(36.9, 37.8)	0.21	< 0.001	37.3(36.9, 37.8)	37.3(36.9, 37.8)	< 0.1	0.214
**Comorbidities, n (%)**								
Myocardial infarct	2172 (14.7)	1540 (22.8)	0.208	< 0.001	1273 (22)	1258 (21.7)	< 0.1	0.753
Congestive heart failure	2984 (20.2)	3161 (46.8)	0.587	< 0.001	2218 (38.3)	2299 (39.7)	< 0.1	0.127
Hypertension	6221 (42.1)	3056 (45.2)	< 0.1	< 0.001	2765 (47.8)	2699 (46.6)	< 0.1	0.226
Hyperlipidaemia	4717 (31.9)	3039 (45)	0.271	< 0.001	2596 (44.8)	2545 (44)	< 0.1	0.35
Peripheral vascular disease	1484 (10)	1115 (16.5)	0.191	< 0.001	887 (15.3)	882 (15.2)	< 0.1	0.918
Dementia	574 (3.9)	355 (5.3)	< 0.1	< 0.001	318 (5.5)	316 (5.5)	< 0.1	0.967
Cerebrovascular disease	2044 (13.8)	1093 (16.2)	< 0.1	< 0.001	868 (15)	924 (16)	< 0.1	0.158
Chronic pulmonary disease	3491 (23.6)	2063 (30.5)	0.156	< 0.001	1684 (29.1)	1686 (29.1)	< 0.1	0.984
Rheumatic disease	501 (3.4)	268 (4)	< 0.1	0.038	228 (3.9)	230 (4)	< 0.1	0.962
Renal disease	2565 (17.4)	1941 (28.7)	0.272	< 0.001	1475 (25.5)	1531 (26.4)	< 0.1	0.244
Peptic ulcer disease	465 (3.1)	191 (2.8)	< 0.1	0.22	176 (3)	174 (3)	< 0.1	0.957
Mild liver disease	2411 (16.3)	657 (9.7)	0.197	< 0.001	596 (10.3)	613 (10.6)	< 0.1	0.627
Severe liver disease	1265 (8.6)	234 (3.5)	0.216	< 0.001	238 (4.1)	231 (4)	< 0.1	0.777
Diabetes	4236 (28.7)	2206 (32.6)	< 0.1	< 0.001	1928 (33.3)	1920 (33.2)	< 0.1	0.89
Malignant cancer	2049 (13.9)	795 (11.8)	< 0.1	< 0.001	747 (12.9)	737 (12.7)	< 0.1	0.802
CCI score	5.0 (3.0, 7.0)	6.0 (5.0, 8.0)	0.493	< 0.001	6.0 (5.0, 8.0)	6.0 (5.0, 8.0)	< 0.1	0.643
**Laboratory parameters**								
Haemoglobin (g/dL)	9.8 (8.4, 11.4)	9.6 (8.2, 11.1)	< 0.1	< 0.001	9.7 (8.3, 11.1)	9.6 (8.3, 11.1)	< 0.1	0.578
Platelets (10^9^/L)	162.0(110.0,227.0)	152.0(109.0,212.0)	< 0.1	< 0.001	157.0(111.0,218.0)	152.0(109.0,213.0)	< 0.1	0.235
WBCs (10^9^/L)	13.8 (10.0, 18.8)	14.2 (10.3, 19.1)	< 0.1	< 0.001	13.7 (10.1, 18.8)	14.3 (10.4, 19.1)	< 0.1	0.002
BUN (mg/dL)	21.0 (14.0, 34.0)	26.0 (17.0, 42.0)	0.203	< 0.001	24.0 (17.0, 39.0)	24.0 (17.0, 40.0)	< 0.1	0.301
Creatinine (mEq/L)	0.9 (0.7, 1.3)	1.0 (0.8, 1.5)	< 0.1	< 0.001	1.0 (0.7, 1.5)	1.0 (0.7, 1.5)	< 0.1	0.224
Glucose (mg/dL)	146.0(118.0,194.0)	142.0(116.0,187.0)	< 0.1	< 0.001	145.0(117.2,191.0)	142.0(116.0,188.0)	< 0.1	0.058
Sodium (mmol/L)	137.0(134.0,140.0)	137.0(135.0,140.0)	< 0.1	0.006	137.0(134.0,140.0)	137.0(135.0,140.0)	< 0.1	0.338
Potassium (mmol/L)	4.4 (4.1, 4.9)	4.5 (4.2, 5.0)	< 0.1	< 0.001	4.5 (4.1, 5.0)	4.5 (4.2, 5.0)	< 0.1	0.056
PTT (s)	32.6 (28.1, 43.9)	35.8 (30.1, 50.1)	0.162	< 0.001	33.6 (28.8, 47.1)	35.3 (29.9, 48.6)	< 0.1	< 0.001
Ph	7.4 (7.4, 7.5)	7.4 (7.4, 7.5)	< 0.1	< 0.001	7.4 (7.4, 7.5)	7.4 (7.4, 7.5)	< 0.1	0.377
po2 (mmHg)	77.0(47.0,112.7)	77.0(46.0,108.0)	< 0.1	0.036	76.6(46.0,110.0)	79.0(47.0,110.0)	< 0.1	0.215
pco2 (mmHg)	46.0(40.0, 54.0)	47.0(41.0, 54.0)	< 0.1	< 0.001	46.8 (40.0, 54.0)	47.0 (41.0, 54.0)	< 0.1	0.044
**Scoring system**								
SOFA score	6.0 (4.0, 8.0)	6.0 (4.0, 9.0)	0.102	< 0.001	6.0 (4.0, 9.0)	6.0 (4.0, 9.0)	< 0.1	0.807
APS III	49.0(35.0, 68.0)	52.0(38.0, 72.0)	0.118	< 0.001	51.0 (37.0, 71.0)	51.0 (37.0, 71.8)	< 0.1	0.677

Abbreviations MAP: mean arterial pressure, CCI: Charlson comorbidity Index, WBCs: leukocyte count, BUN: blood urea nitrogen, pO2: partial pressure of oxygen, pCO2: partial pressure of carbon dioxide, PT: partial thromboplastin time, APS III: Acute Physiology Score III. Note: The chi-square test and Wilcoxon rank-sum test were used to compare the differences in categorical and continuous variables, respectively.

### Characteristics post-PSM

Propensity scores were calculated involving all covariates in Table 1. After 1:1 PSM, 5790 patients in the AF group were matched with 5790 patients in the non-AF group. The degree of imbalance between the two groups was significantly reduced, and the baseline characteristics of the two groups were comparable.

### Outcome comparisons

Before matching, the AF group had a significantly higher 28-day mortality rate, time of mechanical ventilation, ICU LOS and hospital LOS than the non-AF group. After matching, there was little difference between the AF group and the non-AF group in the 28-day mortality rate.

### Evaluation of risk factors for 28-day mortality

**Fig. 3:**
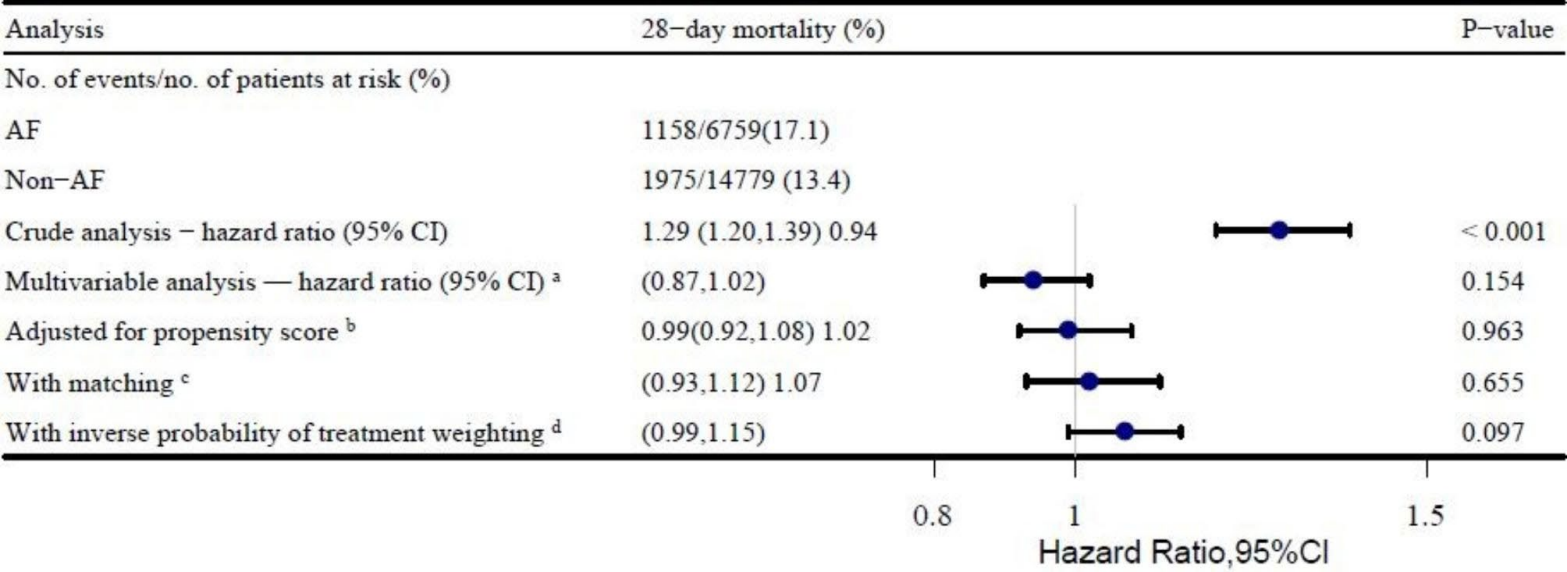
Associations between AF and outcomes in the crude analysis, multivariable analysis, and propensity score analyses. **a.** The hazard ratio was from the multivariable Cox regression model before PSM, with adjustments for all covariates in Table 1. **b.** The hazard ratio was from a multivariable Cox regression model with the same covariates, with additional adjustment for the propensity score. **c.** The hazard ratio was from a multivariable Cox regression model with the same covariates with matching according to the propensity score. The analysis included 11,580 patients (5790 who had AF and 5790 who did not). **d.** The hazard ratio was from the multivariable Cox regression model with the same covariates with inverse probability weighting according to the propensity score

In this study, before PSM, the 28-day mortality rates were 17.1% (1158/6759) and 13.4% (1975/14,779) in the AF and non-AF groups, respectively (Table 2). Univariate Cox regression analysis revealed a significant difference in the 28-day mortality rate (crude HR, 1.29; 95% CI, 1.2–1.39, p < 0.001); after adjusting for all covariates in Table 1, multivariate Cox regression analysis showed that AF was not an independent risk factor for 28-day mortality. AF was not associated with an increased risk of 28-day mortality (HR, 0.94, 95% CI 0.87–1.02, p = 0.154), as shown in Fig. 3. After PSM, the mortality rates for the non-AF and AF groups were 15.6% (905/5790) and 16.1% (933/5790), respectively (Table 2). The results of univariate Cox regression analysis showed that AF was not associated with a higher 28-day mortality rate (HR, 1.02; 95% CI, 0.93–1.12, p = 0.655), as shown in Fig. 3. IPTW analysis demonstrated no difference in the 28-day mortality rate between the non-AF and AF groups. The HR was 1.07 (95% CI, 0.99–1.15, P = 0.097), as shown in Fig. 3. In the sensitivity analyses, similar findings were observed before and after PSM (Supplementary Table 3).

**Table 2:** Clinical outcomes before and after propensity-score matching

Clinical outcomes	Before matching			After matching		
	Non-AF group (n = 14,779)	AF group (n = 6759)	P value	Non-AF group (n = 5790)	AF group (n = 5790)	P value
28-day mortality, n (%)	1975 (13.4)	1158 (17.1)	< 0.001	905 (15.6)	933 (16.1)	0.492
Hospital LOS(d)	8.5 (5.1, 15.3)	9.3 (6.0, 15.6)	< 0.001	8.1 (5.1, 13.9)	9.2 (6.0, 15.7)	< 0.001
ICU LOS(d)	3.0 (1.8, 6.1)	3.5 (2.0, 7.0)	< 0.001	2.9 (1.7, 5.8)	3.4 (1.9, 7.0)	< 0.001
Duration of mechanical ventilation(h)	38.0(16.0,86.0)	49.0(22.0,104.0)	< 0.001	39.0 (18.0, 84.0)	48.0(22.0, 103.0)	< 0.001

LOS: length of stay, ICU: intensive care unit. The chi-square test and Wilcoxon rank-sum test were used to compare the differences in categorical and continuous variables, respectively

### Propensity score matching for secondary outcomes

We evaluated several secondary outcomes after PSM to investigate the effect of AF on the time of mechanical ventilation after ICU admission, ICU LOS and hospital LOS in sepsis patients. First, after ICU admission, the time of mechanical ventilation was significantly longer in the AF group than in the non-AF group (48.0 [22.0, 103.0] vs. 39.0 [18.0, 84.0], respectively, P < 0.001). Second, the ICU LOS and hospital LOS were significantly longer in the AF group than the non-AF group (3.4[1.9–7.0] vs. 2.9[1.7–5.8], respectively, P < 0.001; 9.2[6.0–15.7] vs. 8.1[5.1–13.9], respectively, P < 0.001).

### Kaplan–Meier analysis

**Fig. 4:**
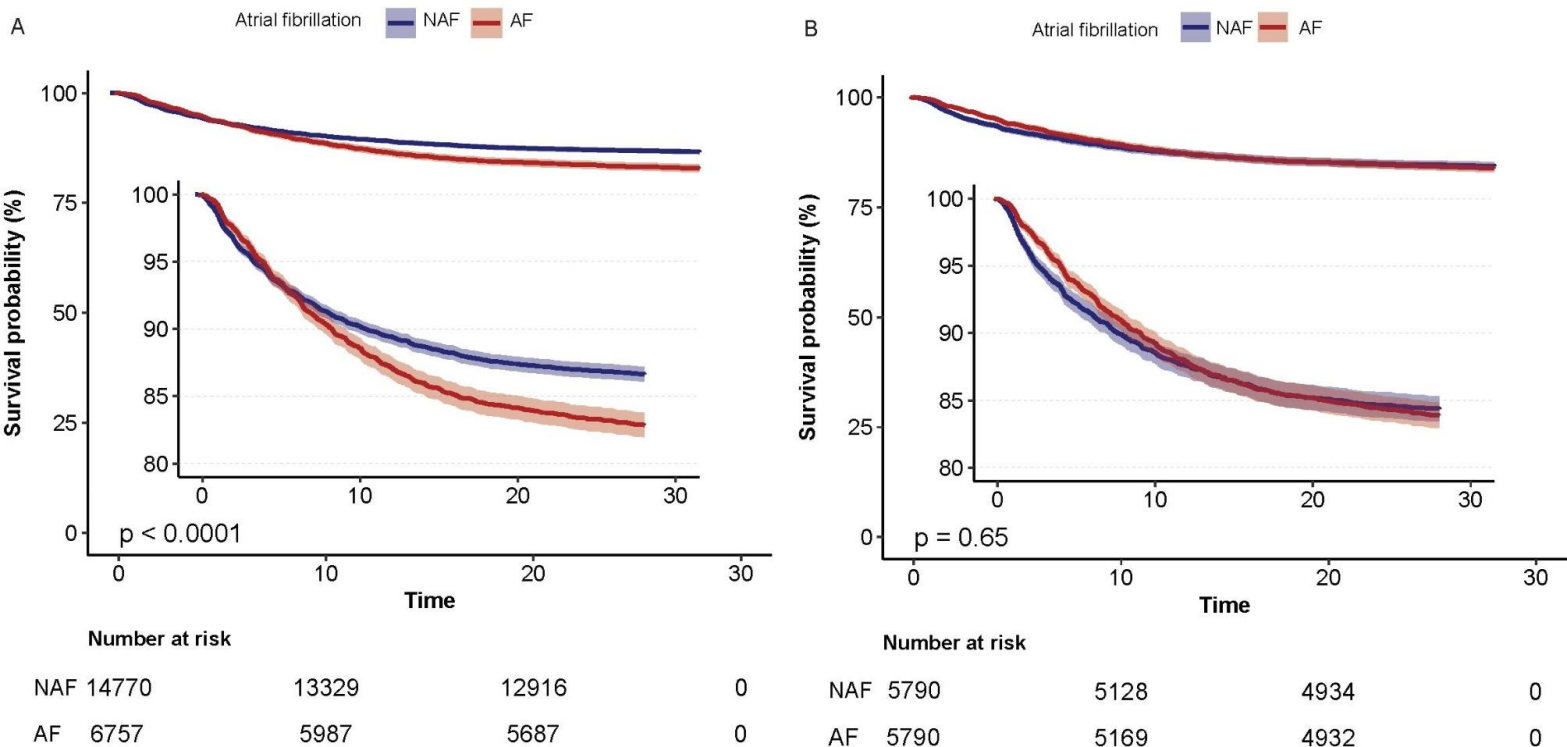
Kaplan–Meier survival curves for survival to 28 days. **(A)** 28-day mortality before propensity score matching; **(B)** 28-day mortality after propensity score matching

Patients were divided into two groups based on whether they had AF on admission. Before PSM, the Kaplan–Meier survival curve analysis showed that the 28-day mortality and survival rates of patients with AF were significantly different from those of patients without AF (log-rank test: p < 0.001) (Fig. 4A). However, there was no difference in 28-day mortality and survival rates between the AF and non-AF groups (log-rank test: p = 0.65) (Fig. 4B) after PSM.

## Discussion

This retrospective cohort study presented data on sepsis patients with and without AF from 2008 to 2019 based on the latest MIMIC-IV database, and the PSM method was used to preprocess the data for causal inference. Little less one-third (31.4%, 6679/21,538) of sepsis patients (first ICU admission) from 2008 to 2019 had AF. We demonstrated that AF might be a risk factor for 28-day mortality among ICU patients with sepsis, but this was inconsistent through multivariate analysis. Furthermore, compared with ICU sepsis patients without AF, those with AF had a higher number of medical comorbidities, a longer ICU LOS and hospital LOS and a longer duration of mechanical ventilation.

AF is the most common arrhythmia in critically ill patients in the ICU^7^. In previous studies, it was reported that patients with AF were more likely to be elderly white males than those without AF, which is in line with our findings [34, 35]. Furthermore, sepsis patients with AF are generally older than those without AF, which is consistent with the findings demonstrated by previous studies [19, 20, 36]. Due to a similar trend with previous studies, this study was sufficiently representative of the population. In addition, this study found that the prevalence of AF in patients with sepsis increased significantly with increasing age. The prevalence increased in a step-by-step manner with increasing age, from 5.1% in those aged under 50 years to 52.4% in those aged over 80 years. A total of 80.9% of sepsis patients with AF in our study were white, which may be caused by differences in medical resource usage [37]. The prevalence of multiple comorbidities was higher in sepsis patients with AF than in those without AF, as reported in previous studies [5, 19, 34, 38]. In our study, sepsis patients with AF had a higher proportion of MI, CHF, hypertension, hyperlipidaemia, PVD, cerebrovascular disease, chronic pulmonary disease, renal disease and DM than those without AF.

Consistent with our results, in a prospective, observational cohort study, Afzal B et al. [24] demonstrated that AF was not a cause of higher mortality in patients with sepsis. Similar to our results, it was observed that AF was independently associated with an increased risk of in-hospital mortality in critically ill patients but was not influenced by the presence of sepsis [9]. Analogous with our results, a multicentre, prospective, inception cohort study found that AF was associated with worse outcomes while not statistically significantly associated with 90-day mortality in ICU patients [39]. Contrary to our study, Salman S et al. [40] found that paroxysmal atrial fibrillation (PAF) was independently associated with 28-day mortality in patients with sepsis by multiple logistic regression analysis and that its development was associated with poor clinical outcomes. Only 81 patients were enrolled in this study, of whom 25 (31%) developed PAF. Desai R et al. [35] conducted a nationwide retrospective cohort study in the United States that included 5,808,166 inpatients with a primary diagnosis of sepsis (19.4% AF) and found that the presence of AF was significantly associated with increased mortality among patients with sepsis-related hospitalizations. The greatest predictors of mortality in this study were race, female sex, older age, and the presence of medical comorbidities. Compared with our study, in the study by Desai R et al., some key risk factors, such as the APS III [41] and laboratory parameters, were not effectively controlled [35]. Klein Klouwenberg et al. conducted a cohort study in two ICUs in the Netherlands (n = 1782) and reported that new-onset AF occurred in 418 (23%) individuals and was independently associated with excess mortality in patients with sepsis [20]. In the present study, patients with sepsis and atrial fibrillation had longer ICU and hospital LOSs and longer durations of mechanical ventilation, even after PSM with adjustment for all covariates included in Table 1; similar results were reported in the study by Christian SA et al. [14.

In this study, there was significant heterogeneity between sepsis patients with and without AF before data preprocessing; hence, the PSM approach was used to address the potential impact of confounding factors. Propensity scores have been proposed as a way to use groups with similar baseline characteristics, especially in studies without randomization. Through random assignment, propensity scores can be thought of as equilibrium scores that try to balance the distribution of measured covariates between two groups [42]. Thus, we applied the PSM approach for all covariates in Table 1 to obtain an equal distribution of generalized conditions and to eliminate heterogeneity between the two groups. Therefore, we adjusted for the detailed covariates between the compared groups using inverse probability weighting to maximize the reduction in bias.

There are several limitations of this study. First, this was a retrospective cohort study, and the diagnosis of sepsis-3 depended merely on administrative diagnosis codes. The diagnostic accuracy could not be directly confirmed by assessing the patients. Thus, misclassifications could lead to false associations. Second, possible selection bias and biases related to unmeasured confounders may exist, as with all retrospective analyses, although we minimized the baseline differences through PSM in this study. Third, considering the nature of the MIMIC database, we lacked data on some potential factors related to the timing of AF diagnosis, the categorization of AF, cardiac function parameters, and the cause of mortality. Finally, all factors (such as the amount of resuscitation fluids administered, the quantitation of catecholamine requirements during shock, or the presence of central venous catheters) may have contributed to the development of AF and/or adversely impacted the outcome parameters measured in this study. There is a need for randomized trials to determine whether the management of AF should be different in sepsis patients and to determine which medications should be preferred to improve prognosis.

## Conclusion

This retrospective study showed that sepsis patients with AF had an increased ICU LOS, increased hospital LOS and increased duration of mechanical ventilation compared with those without AF.

However, after PSM with IPTW and multivariate analyses, it was determined that AF might not be an independent risk factor for sepsis.

### Electronic supplementary material

Below is the link to the electronic supplementary material.

Supplementary Material 1

## Data Availability

The original data used in this study are from the MIMIC-IV database: MIMIC IV (https://physionet.org/content/mimiciv/1.0/, version 1.4). The author (W.W.) obtained access to this database (certification number: 46,543,547) and was responsible for extracting the data. If needed, related data can be requested by contacting W.W.
